# The presence of wingless bat flies *(Eucampsipoda africana)* parasitizing Egyptian fruit bats (*Rousettus aegyptiacus*) in Kagera, Tanzania

**DOI:** 10.1016/j.ijppaw.2026.101306

**Published:** 2026-10-15

**Authors:** Herbertha Elias Mpete, Emma Peter Njau, Mikidadi Issanudia Mtalika, Deusdedith Bwenge Fidelis, Susan Diana Kerfua, Philemon Wambura, Emmanuel George Kifaro

**Affiliations:** a Department of Microbiology, Parasitology and Biotechnology, Sokoine University of Agriculture, Box 3019, SUA, Morogoro, Tanzania; b Tanzania Wildlife Research Institute, Box 661, Arusha, Tanzania; c National Agricultural Research Organization, Box 5704, Kampala, Uganda

**Keywords:** Egyptian fruit bats, *Eucampsipoda africana*, *Rousettus aegyptiacus*, Kagera

## Abstract

Bat ectoparasites are important components of bat communities and have close ecological relationships with their hosts. *Eucampsipoda africana* (*E. africana*) is a wingless bat fly that parasitizes Egyptian fruit bats (*Rousettus aegyptiacus*), but its occurrence and host association have received little attention in Tanzania. A descriptive cross-sectional study was conducted in which 269 bat flies were collected from Egyptian fruit bats and analyzed using morphological identification, mitochondrial cytochrome *c* oxidase subunit I (COI) gene sequencing, and blood-meal analysis. Out of the 193 bats examined, 37.3% (72/193; 95% CI: 30.5-44.5%) of the bats were infested with at least one bat fly. Binary logistic regression showed that age, sex, and village were significantly associated with fly infestation. Adults had higher odds of infestation than juveniles (OR = 4.59, 95% CI: 1.87–11.28), while males had lower odds than females (OR = 0.49, 95% CI: 0.25–0.96). Infestation also differed significantly among villages. COI sequences showed 100% nucleotide identity with reference *E*. *africana* sequences available in GenBank, and phylogenetic analysis clustered the Tanzanian samples with previously reported *E. africana* in Zambia. Blood-meal analysis corroborated the field assumption that these flies have an association with *Rousettus aegyptiacus* (*R. aegyptiacus)*. These findings represent the first molecular confirmation of *E*. *africana* associated with *R*. *aegyptiacus* and of its prevalence in Kagera Region-Tanzania.

## Highlights

• First molecular evidence of *Eucampsipoda africana* (*E. africana*) in Tanzania.
• Morphological identification and COI sequencing confirmed *E. africana.*
• Bat fly blood analysis corroborated field association with *Rousettus aegyptiacus.*
• Tanzanian *E. africana* clustered closely with Zambian isolates.
• Findings established the baseline prevalence of *E. africana* in Tanzania.

## Graphical abstract

**Figure:**
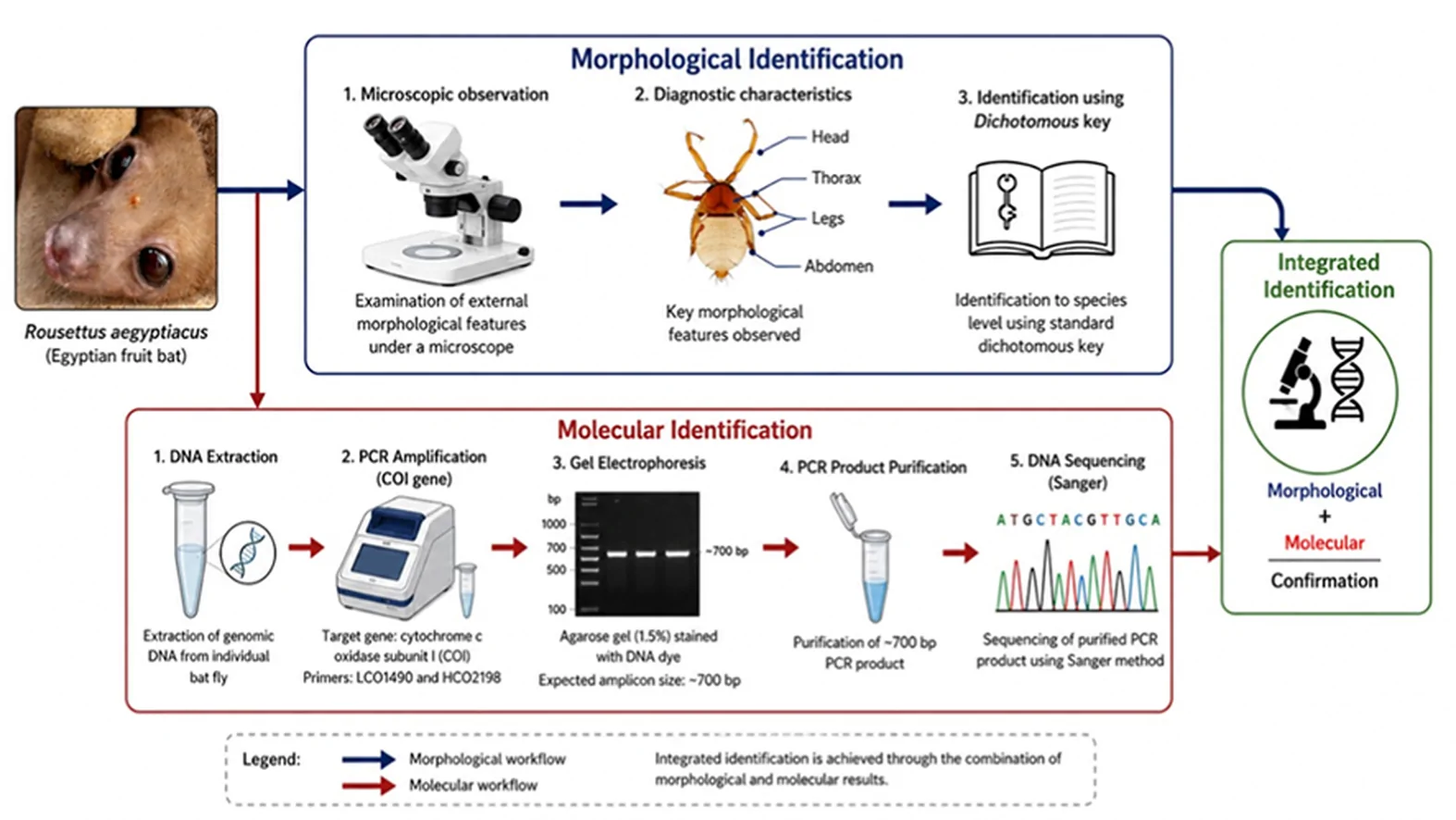

## 1 Introduction

The Egyptian fruit bat *R. aegyptiacus,* is a widely distributed megabat species found throughout sub-Saharan Africa, the Middle East, and parts of the Mediterranean region (Hassanin et al., 2020). Belonging to the family Pteropodidae, these bats typically roost and breed in caves and other underground shelters. Unlike most other plant-eating megabats that roost in tree canopies, *R. aegyptiacus* can navigate dark subterranean environments using a unique form of tongue-click echolocation (Boonman et al., 2013). As primary frugivores (fruit-eaters), they play a vital role in the maintenance and regeneration of tropical forest ecosystems through seed dispersal and cross-pollination (Katunzi et al., 2021). Furthermore, these bats are highly social animals that live in massive colonies, making them essential contributors to cave ecosystems and regional seed dispersal networks.

Bat flies (Diptera: Nycteribiidae and Streblidae) are obligate hematophagous ectoparasites that spend most of their life cycle on their bat hosts (Hiller et al., 2020; Szentiványi et al., 2019). They are among the most host-specific groups of ectoparasites, with many species exhibiting strict one-to-one associations with particular bat species (Dick and Patterson, 2006). This close association makes bat flies valuable models for studying host-parasite coevolution, phylogeography, and host-microbial interactions (Špitalská et al., 2024). In addition to their ecological importance, some bat fly species have been found to harbor bat-associated microorganisms, including Bartonella spp., Polychromophilus parasites, and viruses such as Kanyawara virus, Ledanteviruses, Kaeng Khoi virus, and Wolkberg virus (Billeter et al., 2012; Feng et al., 2017; Qiu et al., 2020).

Among nycteribiid bat flies, *E. africana* is a wingless, eyeless hematophagous ectoparasite of bats (Dick et al., 2023; Goldberg et al., 2017; Lee et al., 2021). *E. africana* is mainly associated with *R. aegyptiacus*, although it has occasionally been reported from other pteropodid bats, particularly where different bat species share the same roosts (Dick et al., 2023; Obame-Nkoghe et al., 2016). They thrive in the warm, humid microclimates of densely populated bat roosts, and females reproduce through adenotrophic viviparity (Çetin et al., 2020; Verrett et al., 2022). *E. africana* has been recorded across sub-Saharan African countries, including Kenya, Uganda, South Africa, and the Democratic Republic of the Congo, although its precise distribution remains poorly documented due to limited surveillance efforts (Goldberg et al., 2017; Theodor, 1967).

Kagera Region provides an important setting for studying bat-associated ectoparasites because it supports several large cave-dwelling colonies of *R*. *aegyptiacus*. Recent surveys identified five major Egyptian fruit bat roosts in caves and mines across Kyerwa, Bukoba Rural, and Muleba districts, with some colonies comprising thousands of individuals (Kinimi et al., 2025). The large colony sizes and frequent contact among bats may facilitate the persistence and host-to-host transfer of ectoparasites such as bat flies, which depend on bats for feeding, reproduction, and survival. Despite the presence of large populations of *R. aegyptiacus* in Kagera Region, information on the occurrence and molecular identity of *E*. *africana* in Tanzania remains limited.

Identifying these ectoparasites on the basis of traditional morphological traits alone is challenging, as many bat flies exhibit highly specialized, reduced features such as winglessness, flattened bodies, and minute appendages that make visual differentiation difficult even under a microscope (Mulvaney et al., 2023). To overcome these limitations, the integration of molecular techniques has become increasingly essential (Pearce et al., 2026). Mitochondrial cytochrome *c* oxidase subunit (COI) gene sequencing is widely recognized as a standard marker for DNA barcoding and enables precise species-level identification, particularly in morphologically reduced taxa such as bat flies (Folmer et al., 1994).

This study aimed to address the limited information on *E*. *africana* parasitizing *R*. *aegyptiacus* in Tanzania by combining morphological and molecular approaches to identify and characterize the species in Kagera Region. The findings provide the first documented record of *E. africana* in Tanzania and extend the known geographical distribution of the species.

## 2 Methodology

### 2.1 Study area

Following the 2023-early 2025 outbreaks of Marburg virus disease (MVD) in the Kagera region, scientific attention has largely focused on bat reservoirs, while the potential contribution of their ectoparasites remains unexplored (Pawęska et al., 2024). Kagera occupies a strategically important ecological and geopolitical position, bordering Uganda, Rwanda, and Burundi and flanked by Lake Victoria to the east (Mmbaga et al., 2024). This transboundary setting, which combines human mobility and wildlife interactions, has increased its epidemiological importance.

This study was conducted in three districts, Kyerwa, Muleba, and Bukoba Rural, within the Kagera Region in northwestern Tanzania, which is a transboundary landscape of significant ecological and epidemiological importance. Kagera lies between approximately 1°00′ and 2°45′ South latitude and 30°25′ and 32°40′ East longitude, occupying the extreme northwest of the United Republic of Tanzania.

The study districts collectively represent a heterogeneous ecological gradient ranging from cave-rich escarpments and forested highlands to humid lowlands dominated by banana and coffee plantations. This varied landscape provides ideal roosting and foraging habitats for Egyptian fruit bats (*R. aegyptiacus*), the recognized natural reservoir of Marburg virus. The ecological diversity, dense bat populations, and recent history of MVD outbreaks make Kagera a critical hotspot for investigating bat-ectoparasite dynamics and their potential role in zoonotic disease ecology.

### 2.2 Study design and sampling strategy

A descriptive cross-sectional study was conducted to investigate the prevalence of bat fly in the Kagera Region, Tanzania. Data were collected from May to June 2025 in three selected districts. Four caves were purposively selected based on their known use as bat roosting sites. The selection was guided by both historical records and information provided by local community members who were familiar with bat populations in the area. Within each village, all accessible caves were systematically identified, mapped, and documented. The selected caves were selectively occupied by *R. aegyptiacus,* with no other bat species observed during the study period. These caves were also regularly accessed by local community members for the collection of bat manure (guano), resulting in routine human movement within the roosting site. The geographic coordinates of each cave were recorded using a Global Positioning System (GPS) device to ensure accurate geolocation of sampling sites. The sampled caves were located in Rugasha Village (Kyerwa District; 36M 241772E, 9883446N), Butahyaibega and Katangalala Villages (Bukoba Rural District; 36M 364830E, 9840122N and 36M 373390E, 9868621N, respectively), and Ibare Village (Muleba District; 36M 346874E, 9807109N).

### 2.3 Sample collection

Bats were captured at cave entrances via mist nets and were handled by trained veterinarians and ecologists wearing full personal protective equipment (PPE) while taking measures to prevent pathogen spillover transmission from bats to humans, biosafety protocols to avoid spillback transmission, and bat welfare measures to minimize the impact on bat populations (Islam et al., 2025). Each bat was briefly and carefully restrained for examination, during which sex, age, and breastfeeding status were recorded. Bat flies were collected via fine-tipped forceps and preserved in 70% ethanol (Barbier and Graciolli, 2016). All the samples were transported at 4 °C via a portable cooling system to the Parasitology Laboratory at Sokoine University of Agriculture (SUA) for storage and analysis.

### 2.4 Morphological identification

Bat flies were removed from 70% ethanol, blotted dry, and morphologically identified via a dissecting microscope at low to medium magnification (up to 40x). Bat fly specimens were examined for key diagnostic external characteristics, including overall body shape, segmentation, head morphology, and appendage structure, particularly the legs (tarsal claws), antennae, and mouthparts (Mlynárová et al., 2023; Theodor, 1967).

### 2.5 Sample preparation for DNA extraction

To enhance DNA yield and facilitate robust PCR amplification, a pooling strategy was adopted. A total of 20 bat flies were selected, comprising five specimens from each of the four study caves. Specimens from the same cave were pooled into a single tube, generating four separate pools aimed at confirming the species-level identity rather than assessing individual genetic diversity**.** These pooled samples were subsequently used for molecular identification of bat flies. Each pool was homogenized with phosphate-buffered saline (PBS) using a sterile mortar and pestle to ensure thorough tissue disruption. All procedures were performed in a class II biological safety cabinet (Telstar company, 8227 Terrassa, Spanien, Spain).

### 2.6 Parasite genome detection for genetic characterization

Genomic DNA was extracted via a commercially available PureLink Genomic DNA Extraction Kit (Thermo Fisher Scientific Company, 168 Third Avenue, Waltham, Massachusetts, 02451, USA) according to the manufacturer's instructions.

Species identification was performed by amplifying the mitochondrial cytochrome *c* oxidase subunit I (COI) gene via polymerase chain reaction (PCR). The forward primer LCO1490 (5′-GGTCAACAAATCATAAAGATATTGG-3′) and the reverse primer HCO2198 (5′-TAAACTTCAGGGTGACCAAAAAACA-3′) were used to amplify an approximately 700 bp fragment (Folmer et al., 1994; Phumee et al., 2025). PCRs were performed in a total reaction volume of 20 μL, containing 5 μL of AllTaq Master Mix 4x, 1 μL of each of the two primers (10 mμ), 11 μL of nuclease-free water, and 2 μL of DNA sample prepared in a PCR tube. The thermocycling conditions were as follows: initial denaturation at 95 °C for 2 min, followed by 40 cycles of denaturation at 95 °C for 5 s, annealing at 52 °C for 15 s, and extension at 72 °C for 30 s, with a final extension at 72 °C for 3 min. Amplified PCR products were resolved on a 1.5% (w/v) agarose gel and stained with Safe-View. Electrophoresis was performed at 110 Voltage for 60 min, followed by visualization under UV illumination.

### 2.7 Host blood meal association

To identify the host species on which the flies had fed, the mitochondrial *cytochrome b* (*cytb*) gene was amplified. The primer sets used to amplify the 623 bp fragment were UNFOR403 (5′-TGAGGACAAATATCATTCTGAGG-3′) and UNREV1025 (5′-GGTTGTCCTCCAATTCATGTTA-3′), as previously described (Folmer et al., 1994; Phumee et al., 2025). The PCR conditions and reaction volumes were identical to those described above, except that annealing was performed at 51 °C. The amplified products were verified on a 1.5% agarose gel as described above.

### 2.8 DNA sequencing for genotyping

PCR products for both the COI and Cytb genes that yielded clear bands of the expected sizes were subjected to Sanger sequencing at Macrogen Europe BV (Amsterdam, The Netherlands). The PCR amplicons were purified and sequenced via a BigDye Terminator Cycle Sequencing Kit (Applied Biosystems, Forest City, CA, USA) with a genetic analyzer (ABI 3730xl, system, Applied Biosystems).

### 2.9 Statistical and molecular sequence analysis

This study adopted a descriptive approach, with molecular techniques used to confirm the species identity of *E. africana*. The occurrence and distribution of *E. africana* were summarized using frequencies and percentages, and the resulting data were analyzed using Microsoft Excel. Also logistic regression models were employed to study the associations between variables such as sex, age, and villages. The differences were considered significant at p-value < 0.05.

Raw DNA sequence data were edited and assembled into consensus sequences via Geneious Prime 2026.0 software. Sequence similarity was assessed via Basic Local Alignment Search Tool for Nucleotide (BLASTn) searches against the NCBI GenBank database. Multiple sequence alignments were performed in Molecular Evolutionary Genetics Analysis (MEGA) version X. Phylogenetic analyses were conducted via the maximum likelihood method with 1000 bootstrap replicates under the Kimura 2-parameter model (Kimura, 1980; Kumar et al., 2018).

## 3 Results

### 3.1 Bat fly infestation rate

A total of 193 *R. aegyptiacus* bats were captured, comprising 103 males and 90 females. Of these, 72 (37.3%) were infested with bat flies, while 121 (62.7%) were uninfested, resulting in an overall infestation prevalence of 37.3% (72/193; 95% CI: 30.5–44.5%). A total of 269 bat flies were collected from bats sampled in Kyerwa, Bukoba Rural, and Muleba districts of Kagera Region. This corresponded to a mean abundance of 1.39 bat flies per examined bat and a mean intensity of 3.74 bat flies per infested bat. Of the 269 bat flies collected, 106 (39.4%) contained visible blood in their digestive tracts. Infestation prevalence varied across the study villages, with the highest prevalence recorded in Butahyaibega (70.6%), followed by Rugasha (37.3%), Katangalala (26.8%), and Ibare (20.9%). Female bats had a higher infestation prevalence (47.8%, 43/90) than males (28.2%, 29/103). Among the 18 lactating females captured, six (33.3%) were infested with bat flies (Table 1).

**Table 1:** Distribution of bat fly infestation, sex, age group, and prevalence by village.

Village	Bats captured	Sex		Bats with flies		Age		Lactating females	Infestation rate
		Male	Female	Male	Female	Adult	Juvenile		
Rugasha	75	31	44	9	19	34	41	7	37.3%
Butahyaibega	34	16	18	9	15	30	4	4	70.6%
Katangalala	41	28	13	7	4	40	1	5	26.8%
Ibare	43	28	15	4	5	39	4	2	20.9%
Total	193	103	90	29	43	143	50	18	37.3%

Binary logistic regression identified age, sex, and village as significant predictors of bat fly infestation in *R. aegyptiacus* (Table 2). Adult bats had significantly higher odds of infestation than juveniles (OR = 4.59, 95% CI: 1.87–11.28, p = 0.0009), indicating that they were approximately 4.6 times more likely to be infested. Male bats had 51% lower odds of infestation than females (OR = 0.49, 95% CI: 0.25–0.96, p = 0.0377), indicating that females were more likely to be infested. Compared with bats from Butahyaibega, the odds of infestation were significantly lower among bats from Ibare (OR = 0.11, 95% CI: 0.04–0.32, p = 0.0001), Katangalala (OR = 0.18, 95% CI: 0.07–0.52, p = 0.0014), and Rugasha (OR = 0.10, 95% CI: 0.04–0.30, p < 0.0001), demonstrating significant spatial variation in infestation across the sampled villages.

**Table 2:** Factors associated with *E. africana* infestation among bats using logistic regression.

Variable	Odds Ratio	95% CI	Coefficient (β)	SE	Z-Statistic	P-value	
Age group (Juvenile vs Adult)	4.59	1.87–11.28	1.524	0.458	3.325	0.0009	*
Sex (Male vs Female)	0.49	0.25–0.96	−0.708	0.341	−2.078	0.0377	*
Village (Ibare vs Butahyaibega)	0.11	0.04–0.32	−2.212	0.553	−3.998	0.0001	*
Village (Katangalala vs Butahyaibega)	0.18	0.07–0.52	−1.695	0.530	−3.202	0.0014	*
Village (Rugasha vs Butahyaibega	0.1043	0.04-0.03	−2.264	0.542	−4.167	0.0001	*

**Note:** * Statistically significant.

### 3.2 Morphological confirmation

Morphological examination identified that all collected bat flies as a single morphospecies, *E. africana,* (Fig. 1), based on morphological features consistent with published descriptions (Mlynárová et al., 2023; Theodor, 1967).

**Fig. 1:**
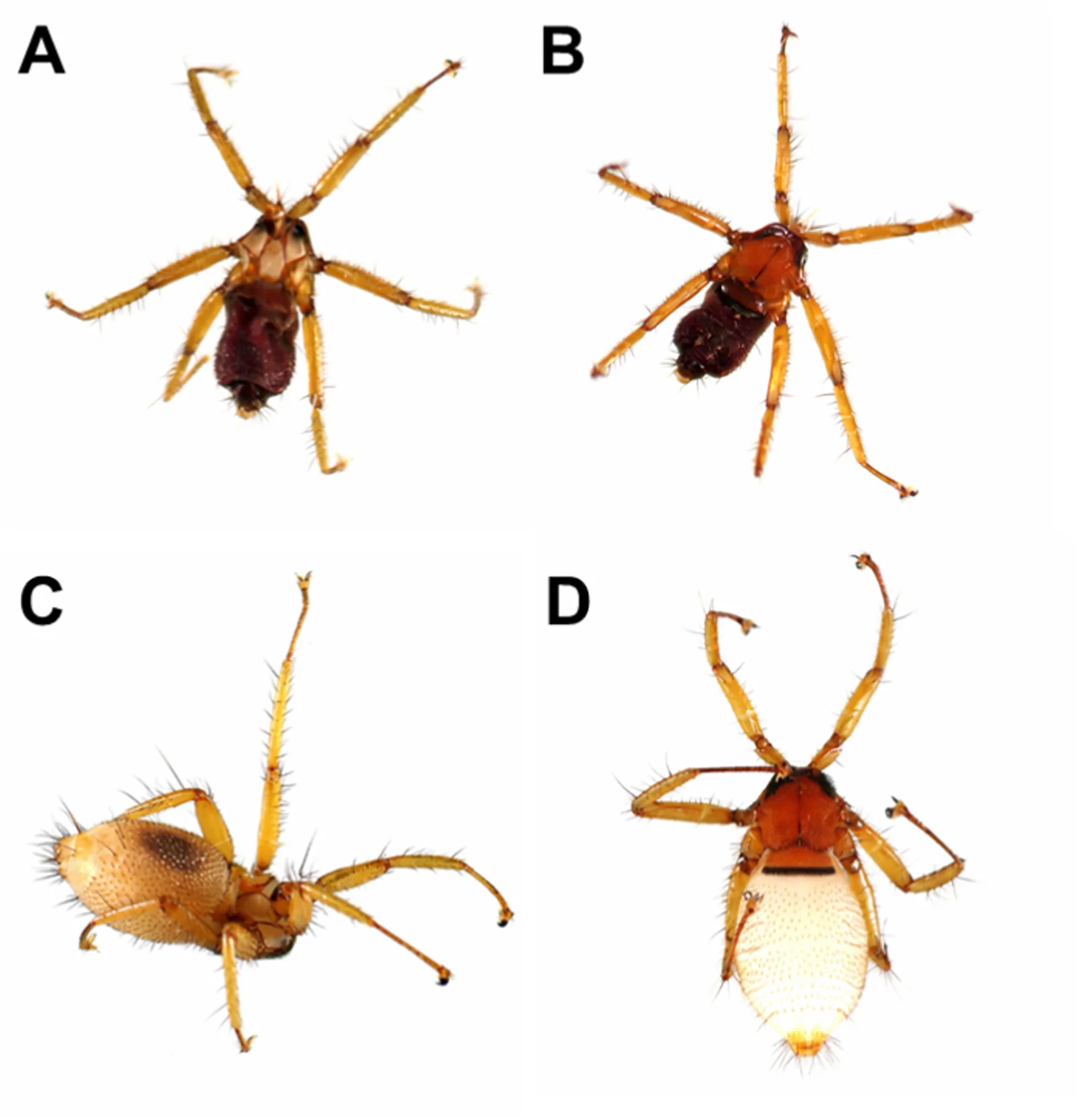
Images of *E. africana* collected in the Kagera region. A&C represents Dorsal view of *E. africana,* while B&D represents ventral view of *E. africana.*

### 3.3 Molecular and phylogenetic analysis of bat flies

All bat fly samples yielded successful PCR amplification of the cytochrome oxidase subunit I (COI) gene, which produced amplicons of the expected size (∼700 bp). The obtained sequences were compared with reference sequences available in GenBank (Table 3). Molecular analysis of COI sequences from representative labeled samples (Kagera/Tan/S1/2025 and Kagera/Tan/S2/2025) revealed 100% nucleotide identity with *E. africana* sequences deposited in GenBank (accession number LC536588). Phylogenetic analysis revealed that all sequences generated from bat flies collected in the Kagera region clustered with *E. africana* isolates previously reported from Zambia (Fig. 2).

**Table 3:** List of species and GenBank accession numbers of sequences used for bat fly species identification.

Voucher	Species	Host	Country	Accession number	Reference
ZBF2	*E. africana*	*R. aegyptiacus*	Zambia	LC536587	Qiu et al., 2020
ZBF1	*E. africana*	*Rousettus aegyptiacus*	Zambia	LC536586	Qiu et al., 2020
NG13-013-2	*E. africana*	*Rousettus aegyptiacus*	Nigeria	MH151066	(Bai et al., 2018)
NG13-018	*E. africana*	*Rousettus aegyptiacus*	Nigeria	MH151065	(Bai et al., 2018)
MIZ475-EM-F27A	*Eucampsipoda madagascarensis*	*Rousettus madagascariensis*	Madagascar	OR732305	(Andrianiaina et al., 2025)
J48	*Eucampsipoda madagascarensis*	*Rousettus madagascariensis*	Madagascar	KF021495	Tortosa et al.*,* 2013
J43	*Eucampsipoda madagascarensis*	*Rousettus madagascariensis*	Madagascar	KF021494	Tortosa et al.*,* 2013
N8	*Eucampsipoda inermis*	*Rousettus amplexicaudatus*	Philippines	KF021493	Tortosa et al.*,* 2013
KB5	*Eucampsipoda sundaica*	*Pteropus vampyrus*	Malaysia	KF273783	(Olival et al., 2013)
8B	*Eucampsipoda theodori*	*Rousettus obliviosus*	Comoros	KF021500	Tortosa et al.*,* 2013
28DM	*Eucampsipoda theodori*	*Rousettus obliviosus*	Comoros	KF021496	Tortosa et al.*,* 2013
BF801	*Dipseliopoda* sp	*Myonycteris angolensis*	Uganda	MT278273	Bennett et al.*,* 2020
BF301	*Dipseliopoda* sp	*Myonycteris angolensis*	Uganda	MT278270	Bennett et al.*,* 2020
**Kagera/Tan/S1/2025**	***Eucampsipoda africana***	***Rousettus aegyptiacus***	**Tanzania**	**PZ514107**	**This study**
**Kagera/Tan/S2/2025**	***Eucampsipoda africana***	***Rousettus aegyptiacus***	**Tanzania**	**PZ514108**	**This study**

**Fig. 2:**
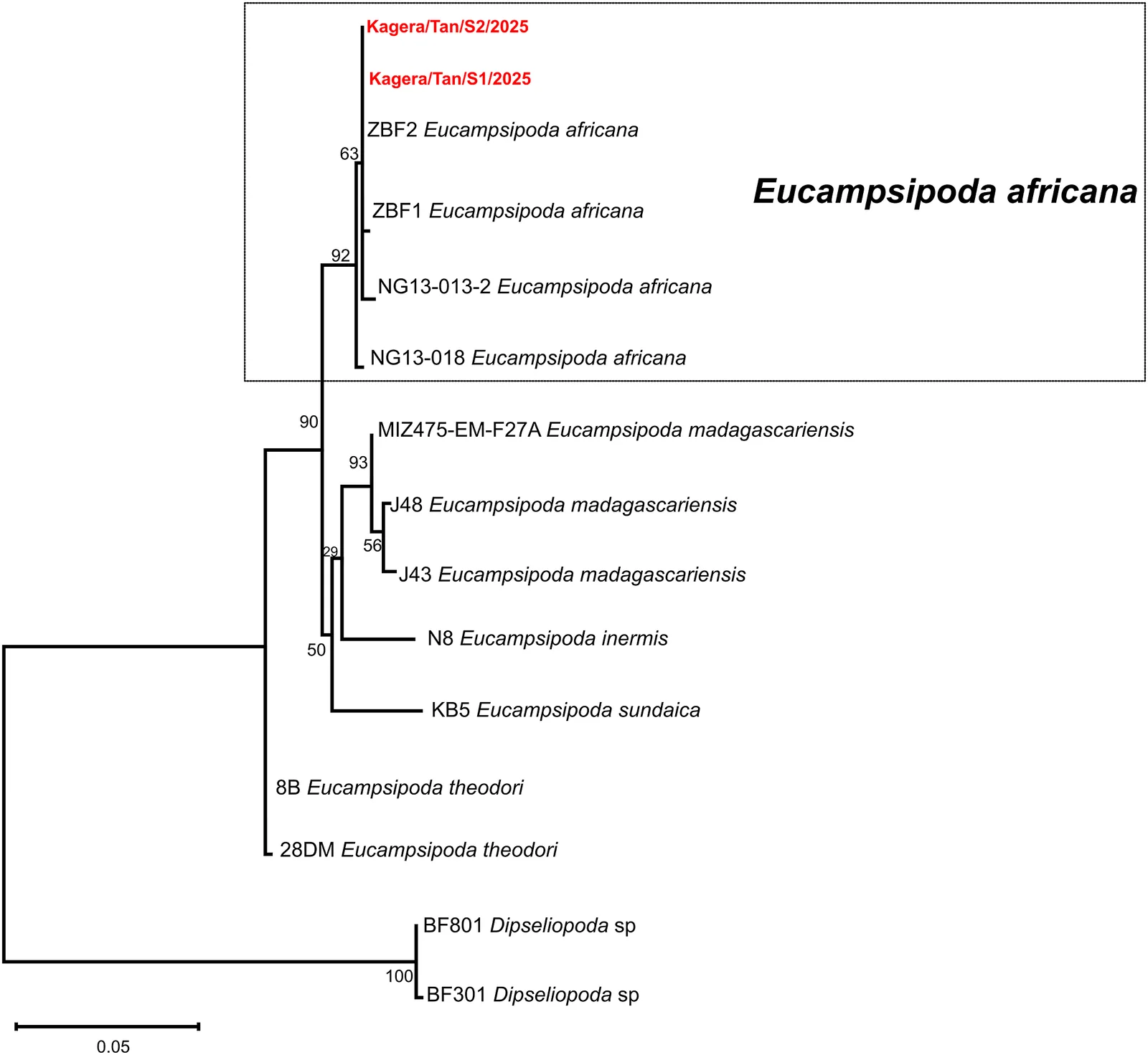
Maximum-likelihood phylogenetic tree of bat flies based on mitochondrial COI sequences. Branches are annotated with maximum likelihood bootstrap values. Bat fly sequences generated in this study are in bold red.

### 3.4 Host blood meal association

*Cytochrome b (Cytb)* PCR was used to confirm the mammalian host feeding association with bat fly. All tested bat fly pools yielded bat-derived sequences. BLAST results revealed 98% identity and 100% query coverage with reference sequences in GenBank, confirming *R. aegyptiacus* as the host species (Table 4). Phylogenetic analysis of the Cytb sequences corroborated the BLAST results, with all sequences obtained in this study clustering within the *R. aegyptiacus* clade, confirming host association. (Fig. 3).

**Table 4:** List of species and GenBank accession numbers of sequences used for host blood meal association.

Voucher	Species	Country	Accession number	Reference
**GD36**	*Rousettus leschenaultia*	China	FJ549324	Direct submission
**T265**	*Rousettus leschenaultia*	Central Africa	KX823315	(Hassanin et al., 2016)
**9407**	*Rousettus aegyptiacus*	Guinea	OQ383610	Makenov et al.*,* 2023
**NMK187164**	*Rousettus aegyptiacus*	Kenya	MN064745	(Lutz et al., 2019)
**S63247**	*Rousettus aegyptiacus*	Central Africa	KX822963	(Hassanin et al., 2016)
**S63313**	*Rousettus aegyptiacus*	Central Africa	KX822973	(Hassanin et al., 2016)
**C07X140**	*Rousettus aegyptiacus*	Central African	JF728760	(Nesi et al., 2011)
**USNM449206**	*Rousettus madagascariensis*	Madagascar	JN398214	(Almeida et al., 2011)
**FMNH 187670**	*Rousettus madagascariensis*	Madagascar	GU228597	(Goodman et al., 2010)
**FMNH 194543**	*Rousettus obliviosus*	Comoros	GU228752	(Goodman et al., 2010)
**FMNH 194234**	*Rousettus obliviosus*	Comoros	GU228729	(Goodman et al., 2010)
**MNHN 2014-564**	*Scotonycteris bergmansi*	Cameroon	KP306495	(Hassanin et al., 2015)
**MNHN 2011-713**	*Scotonycteris bergmansi*	Cameroon	KP306494	(Hassanin et al., 2015)
**MNHN 2011-675**	*Scotonycteris zenkeri*	Cameroon	KP306470	(Hassanin et al., 2015)
**MNHN 2014-565**	*Scotonycteris zenkeri*	Cameroon	KP306466	(Hassanin et al., 2015)
**MNHN:2011-637**	*Casinycteris campomaanensis*	Southwestern Cameroon	KJ145797	Hassanin et al.*,* 2014
**MNHN 2011-683**	*Casinycteris argynnis*	Cameroon	KP306421	(Hassanin et al., 2015)
**T670**	*Stenonycteris lanosus*	Tanzania	JX283245	(Nesi et al., 2013)
**G51699**	*Hypsignathus monstrosus*	Central Africa	KX823021	(Hassanin et al., 2016)
**T310**	*Epomophorus crypturus*	Central Africa	KX823308	(Hassanin et al., 2016)
**K09224**	*Epomophorus anselli*	Central Africa	KX823307	(Hassanin et al., 2016)
**T217**	*Micropteropus pusillus*	Central African Republic	JF728672	(Nesi et al., 2011)
**C7151**	*Nanonycteris veldkampii*	Central Africa	KX822985	(Hassanin et al., 2016)
**ROM100560**	*Nanonycteris veldkampii*	Royal Ontario Museum	JN398209	(Almeida et al., 2011)
**G09108**	*Megaloglossus azagnyi*	Ivory Coast	JX283271	(Nesi et al., 2013)
**A9**	*Megaderma lyra*	China	DQ888678	(Li et al., 2007)
**ROM 110979**	*Taphozous melanopogon*	Vietnam	EF584221	(Lim et al., 2007)
**Kagera/Tan/BB1/2025**	***Rousettus aegyptiacus***	**Tanzania**	**PZ513219**	**This study**
**Kagera/Tan/BF1/2025**	***Rousettus aegyptiacus***	**Tanzania**	**PZ513220**	**This study**
**Kagera/Tan/BF2/2025**	***Rousettus aegyptiacus***	**Tanzania**	**PZ513221**	**This study**

**Fig. 3:**
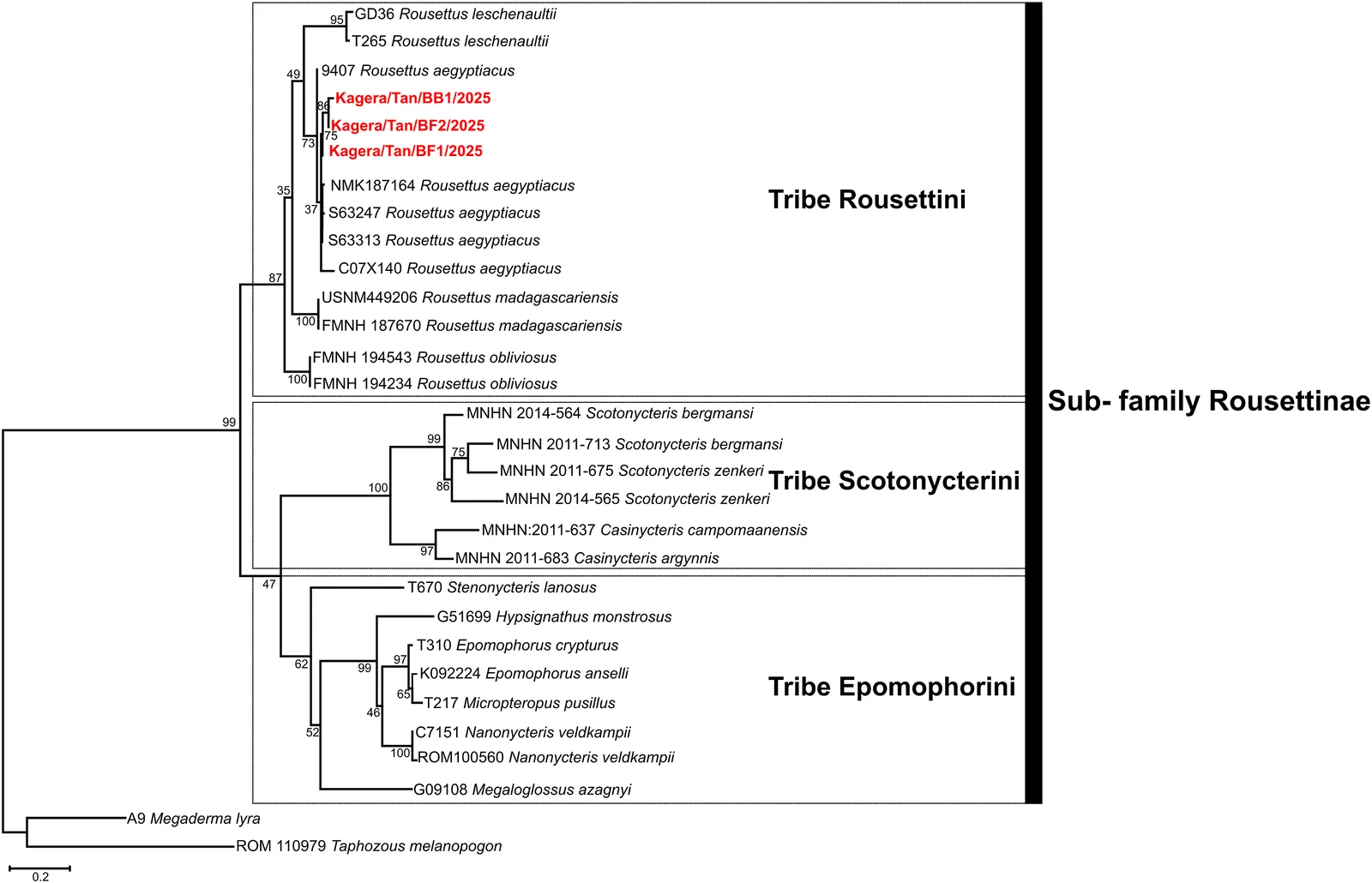
Maximum-likelihood phylogenetic tree of fruit bats based on mitochondrial cyt *b* sequences. Branches are annotated with maximum likelihood bootstrap values. The fruit bat sequences generated in this study are shown in bold red.

## 4 Discussion

The overall prevalence of bat fly infestation in this study was 37.3%, showing that bat flies were present in more than one-third of the *R. aegyptiacus* examined. This finding was similar to the 38.0% infestation reported among cave-dwelling bats in Gabon, where *E. a fricana* was also recorded from *R. aegyptiacus* (Obame-Nkoghe et al., 2016). However, the prevalence observed in the present study was lower than the 95.2–100% reported for *E. africana* on *R. aegyptiacus* in South Africa (Pawęska et al., 2021). Differences in infestation prevalence between studies may be related to variation in host populations, sampling periods, roost conditions, and other local factors. Nevertheless, the finding indicates that bat fly infestation was relatively widespread among the sampled *R. aegyptiacus* population in Kagera Region.

Beyond the overall prevalence, infestation varied with host age. Adults had approximately 4.6 fold higher odds of fly infestation than juveniles, which may be related to their longer period of exposure to bat flies and repeated contact within shared roosts. Similar age-related patterns have been reported in other bat ectoparasite studies, where adult bats carried higher parasite burdens than juveniles (Atobatele et al., 2023; Lee et al., 2018; Lim et al., 2020; Rajemison et al., 2017). These findings suggest that greater cumulative exposure may partly explain the higher odds of infestation observed among adults in the present study, although age-related patterns can vary among host–parasite systems.

Sex was also significantly associated with bat fly infestation, with male *R. aegyptiacus* having 51% lower odds of infestation than females. The higher infestation observed among females may be related to differences in social and roosting behavior, as closer contact among individuals could provide more opportunities for bat flies to move between hosts. Similar female-biased ectoparasitism has been reported in other bat studies (Bezerra and Bocchiglieri, 2023; Patterson et al., 2008). However, this finding differs from a study in South Africa, where male *R. aegyptiacus* had higher *E. africana* infestation (Pawęska et al., 2021). The contrasting findings may reflect differences in host behavior, population characteristics, or sampling conditions between the study areas.

In addition to host-related factors, village was significantly associated with bat fly infestation, indicating that infestation varied among the sampled locations. Differences in bat fly occurrence among locations have been linked to variation in bat colonies, roosting patterns, and host–parasite interactions (Novianto et al., 2025; Saldaña-Vázquez et al., 2019). Studies of *E. africana* associated with *R. aegyptiacus* have also reported variation in infestation under different local host and roost conditions (Obame-Nkoghe et al., 2016; Pawęska et al., 2021). In the present study, the observed differences among villages may partly reflect human activities around the caves, particularly farming and bat manure collection, may influence how bats use these roosts and could therefore contribute to differences in infestation.

Morphological characteristics and mitochondrial cytochrome *c* oxidase subunit I (COI) gene sequencing confirmed the presence of *E. africana*. The Tanzanian sequences showed 100% nucleotide identity with a previously reported *E. africana* sequence and clustered closely with *E. africana* sequences from Zambia in the phylogenetic analysis. This genetic similarity suggests that geographically separated *E. africana* populations may share closely related mitochondrial lineages (Qiu et al., 2020). Such patterns could be influenced by the distribution and occasional movements of *R. aegyptiacus*, which can facilitate dispersal of their wingless ectoparasitic bat flies and contribute to gene flow among fly populations (McKee et al., 2024). However, broader geographic sampling and the use of additional genetic markers are needed to elucidate the population structure and evolutionary relationships of *E. africana* across Africa.

The detection of *R. aegyptiacus* DNA in the examined bat flies further supports their association with Egyptian fruit bats. This finding is consistent with the well-documented association between *E. africana* and *Rousettus* bats across several African regions (McKee et al., 2024; Seneviratne et al., 2009; Szentiványi et al., 2019). However, the detection of host DNA is interpreted as evidence of recent feeding rather than definitive evidence of strict host specificity. In the present study, the flies were collected directly from *R. aegyptiacus*, and the detection of host DNA is therefore consistent with this bat serving as a host. Nevertheless, analysis of individual flies and sampling of other bat species would be required to determine whether *E. africana* preferentially utilizes *R. aegyptiacus* or can exploit other bat hosts. Such studies would also help clarify whether host associations vary geographically.

The detection and molecular confirmation of *E. africana* on *R. aegyptiacus* in Kagera Region adds important baseline information on the occurrence of this bat fly in Tanzania. By combining morphological identification with COI sequence analysis, this study provides the first molecular evidence of *E. africana* in the country and helps address the limited information currently available on bat flies in Tanzania. These findings provide a useful foundation for future studies investigating the distribution, host associations, and genetic diversity of *E. africana* across different bat populations in Tanzania.

## 5 Conclusion

This study provides the first molecular confirmation of *E. africana* associated with *R. aegyptiacus* in Tanzania, expanding current knowledge of Nycteribiidae distribution in the country. By combining morphological and molecular identification, it establishes an important baseline for future research on the distribution, host associations, and genetic diversity of bat flies in Tanzania. Given that *R. aegyptiacus* is a recognized reservoir of zoonotic pathogens, understanding its associated ectoparasites is an important step toward improving knowledge of bat–parasite interactions, even though pathogen screening was beyond the scope of this study.

## 6 Recommendations

Future studies should incorporate pathogen screening, including the use of viral and bacterial agents, to assess the potential role of *E. africana* in pathogen maintenance and transmission within bat colonies. Expanded sampling across additional regions of Tanzania and neighboring countries is recommended to evaluate the genetic diversity, population structure, and regional connectivity of bat fly populations. Longitudinal studies examining seasonal dynamics, reproductive status, and roosting ecology could further clarify the factors influencing infestation patterns. Finally, integrating ecological, molecular, and epidemiological approaches will enhance the understanding of bat–ectoparasite–microbe networks and inform wildlife health surveillance and zoonotic risk assessments in East Africa.

## CRediT authorship contribution statement

**Herbertha Elias Mpete:** Writing – review & editing, Writing – original draft, Methodology, Formal analysis, Data curation, Conceptualization. **Emma Peter Njau:** Supervision, Conceptualization. **Mikidadi Issanudia Mtalika:** Investigation. **Deusdedith Bwenge Fidelis:** Investigation. **Susan Diana Kerfua:** Investigation. **Philemon Wambura:** Supervision. **Emmanuel George Kifaro:** Supervision, Project administration, Funding acquisition, Conceptualization.

## Ethical approval

Research clearance for sampling Egyptian fruit bats in the Kagera region was granted by the Joint Management Research Committee (JMRC) of the Tanzania Wildlife Research Institute (TAWIRI), Reference Number: AB.235/325/01″ A”/50 of 12 February 2025; and the Tanzania Commission for Science and Technology (COSTECH), Reference Number: CST00001167-2025-2025-00290, issued on 19 March 2025. Consent to participate is not applicable.

## Consent for publication

Not applicable.

## Availability of data and material

The nucleotide sequences generated in this study have been deposited in GenBank under accession numbers PZ513219, PZ513220, PZ513221, PZ514107, and PZ514108.

## Funding

This work was supported by the Partnership for Skills in Applied Sciences, Engineering and Technology (PASET), the Regional Scholarship and Innovation Fund (RSIF), the Junior Investigator Research Award (JIRA), and Grant No. RSIF/JIRA/009.

## Declaration of conflict of interest

The authors declare that they have no conflict of interests to disclose.

## List of abbreviations

COI: Mitochondrial Cytochrome C oxidase subunit I gene
Cytb: Mitochondrial cytochrome *b*
BLASTn: Basic local alignment search tool for nucleotides
PPE: Personal protective equipment
MVD: Marburg virus disease
DNA: Deoxyribonucleic acid
PCR: Polymerase chain reaction
